# Targeting Receptor Tyrosine Kinases for Chemoprevention by Green Tea Catechin, EGCG

**DOI:** 10.3390/ijms9061034

**Published:** 2008-06-20

**Authors:** Masahito Shimizu, Yohei Shirakami, Hisataka Moriwaki

**Affiliations:** Department of Medicine, Gifu University Graduate School of Medicine, Gifu 501-1194, Japan

**Keywords:** Tea catechins, EGCG, cell signaling pathway, RTK, AP-1, activator protein-1, COX-2, cyclooxygenase-2, EC, (–)-epicatechin, ECG, epicatechin-3-gallate, EGC, (–)-epigallocatechin, EGCG, (–)-epigallocatechin-3-gallate, EGF, epidermal growth factor, EGFR, epidermal growth factor receptor, ERK, extracellular signal-regulated kinase, FGF, fibroblast growth factor, FGFR, fibroblast growth factor receptor, HNSCC, head and neck squamous cell carcinoma, IGF-1, insulin-like growth factor-1, IGF-1R, insulin-like growth factor-1 receptor, IGFBP, insulin-like growth factor-binding protein, IκBα, inhibitor of κB-α, IKKα, inhibitor of κB kinase-α, LR, laminin receptor, MAPK, mitogen-activated protein kinase, MEK, mitogen-activated protein kinase kinase, MMP, matrix metalloproteinase, PDGF, platelet-derived growth factor, PDGFR, platelet-derived growth factor receptor, PGE_2_prostaglandin E_2_, Poly E, polyphenon E, PI3K, phosphatidylinositol 3-kinase, ROS, reactive oxygen species, RTK, receptor tyrosine kinase, Stat, signal transducers and activator of transcription, TGFα, transforming growth factor-α, TRAMP, transgenic adenocarcinoma of mouse prostate, UV, ultraviolet, VEGF, vascular endothelial growth factor, VEGFR, vascular endothelial growth factor receptor

## Abstract

Tea is one of the most popular beverages consumed worldwide. Epidemiologic studies show an inverse relationship between consumption of tea, especially green tea, and development of cancers. Numerous *in vivo* and *in vitro* studies indicate strong chemopreventive effects for green tea and its constituents against cancers of various organs. (–)-Epigallocatechin-3-gallate (EGCG), the major catechin in green tea, appears to be the most biologically active constituent in tea with respect to inhibiting cell proliferation and inducing apoptosis in cancer cells. Recent studies indicate that the receptor tyrosine kinases (RTKs) are one of the critical targets of EGCG to inhibit cancer cell growth. EGCG inhibits the activation of EGFR (erbB1), HER2 (neu/erbB2) and also HER3 (neu/erbB3), which belong to subclass I of the RTK superfamily, in various types of human cancer cells. The activation of IGF-1 and VEGF receptors, the other members of RTK family, is also inhibited by EGCG. In addition, EGCG alters membrane lipid organization and thus inhibits the dimerization and activation of EGFR. Therefore, EGCG inhibits the Ras/MAPK and PI3K/Akt signaling pathways, which are RTK-related cell signaling pathways, as well as the activation of AP-1 and NF-κB, thereby modulating the expression of target genes which are associated with induction of apoptosis and cell cycle arrest in cancer cells. These findings are significant because abnormalities in the expression and function of RTKs and their downstream effectors play a critical role in the development of several types of human malignancies. In this paper we review evidence indicating that EGCG exerts anticancer effects, at least in part, through inhibition of activation of the specific RTKs and conclude that targeting RTKs and related signaling pathway by tea catechins might be a promising strategy for the prevention of human cancers.

## 1. Introduction

Many epidemiologic and laboratory studies have demonstrated accumulating evidence that a diet which has a high content of fruits and vegetables reduces the risk for several types of cancer [1, 2]. These findings suggest that, in the future, the use of nutritional supplements derived from fruits and vegetables will be a useful strategy for the prevention of cancer. However, the specific components of these foodstuffs and the precise mechanisms that exert this protective effect have not yet been thoroughly elucidated. In recent studies, a number of specific phytochemicals, non-nutritive components in the plant-derived diets, have been identified which possess anti-carcinogenic and anti-mutagenic effects in various experimental systems [2]. The cellular and molecular mechanisms by which specific phytochemicals cause such an anti-carcinogenic effect remain to be clarified, including the antioxidant activity and/or trapping of oxygen radicals, the induction of drug metabolizing and detoxifying enzymes, the promotion of DNA repair, and controlling the expression and functional activity of tumor-suppressor genes, etc [2–4]. In addition to these mechanisms, recent studies have demonstrated that phytochemicals exert an anti-carcinogenic effect by modulating the activities of various types of receptor tyrosine kinases (RTKs) and their downstream specific cell signaling pathways which are associated with the expression of the genes involved in cell proliferation and apoptosis [2, 5–8].

**Figure 1.**
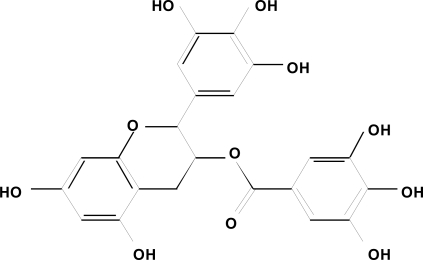
Chemical structure of EGCG.

Among candidate phytochemicals, (–)-epigallocatechin gallate (EGCG; Figure 1), the major biologically active component in green tea, seems to be one of the most potent polyphenols with respect to inhibiting cell proliferation and inducing apoptosis in cancer cells [5, 6, 9]. The anti-proliferative effects of EGCG are partially explained by their antioxidative properties [9]. In addition, recent investigators have demonstrated EGCG to exhibit several novel mechanisms which inhibit the growth of cancer cells. For instance, we reported that EGCG exerts its anti-tumor effect by inhibiting the activation of some types of RTKs and their multiple downstream signaling pathways in human head and neck squamous cell carcinoma (HNSCC), breast cancer, colorectal cancer, and liver cancer cells [5, 10–16]. EGCG also inhibits the binding of EGF to the EGFR, which belong to subclass I of the RTK superfamily, thereby blocking the subsequent dimerization and activation of this receptor by altering membrane organization [17]. These findings seem to be of great significance because the cell surface RTKs are now attracting a great deal of attention as a specific targets of the “molecular-targeted therapy” for cancer [18]. Therefore, this review focuses on the novel and updated mechanisms by which EGCG prevents carcinogenesis, with a special focus on the effects of this agent on the activation of RTKs, especially EGFR, HER2, HER3, and IGF-1R, because abnormalities in the expression and/or activation of these receptors and their ligands have been reported to play a critical role in the development of human malignancies [19, 20]. This article also reviews the effects of EGCG on the central components of the complex intracellular network of signal transduction, including the Ras/MAPK and PI3K/AKT pathways, which are located in the downstream of RTK signaling [21, 22]. The targeting of these signaling pathways may thus provide an effective strategy for the prevention and treatment of cancer because during the process of carcinogenesis, abnormalities in these pathways and/or the related downstream transcription factors are known to cause uncontrolled cell replication and malignant cell transformation [23,24].

## 2. Green Tea and Cancer Chemoprevention

Tea, especially green tea, produced from the leaves of the *Camellia sinensis*, is one of the most widely consumed beverages in the world. Green tea contains several polyphenolic compounds, including the EGCG, (–)-epigallocatechin (EGC), epicatechin-3-gallate (ECG), and (–)-epicatechin (EC). It is widely accepted that the consumption of green tea has a beneficial effect on health, including a chemopreventive efficacy against various types of cancer. Therefore, in recent years, several epidemiological studies as well as studies in rodent and *in vitro* models have shown that green tea and its main polyphenol, EGCG, can provide protection against various malignancies including skin, breast, prostate, lung, colon, liver, stomach, and other types of cancers [5, 9, 25]. As briefly described in the “Introduction” section, the anti-cancer effect of green tea and its polyphenolic catechins is partially attributed to the antioxidative properties and the ability to scavenge reactive oxygen species (ROS) [9]. The phenolic hydroxy groups that exist in tea catechins play an important role in antioxidant and radical scavenging activity [26]. In addition, green tea and EGCG have been shown to exert both anti-angiogenic [27, 28] and anti-mutagenic effects [29]. The anti-inflammatory activity of green tea and EGCG is also exerts a chemopreventive effect because inflammation is a risk factor for most type of cancers [11, 30–32]. These diverse effects of green tea and EGCG may therefore play a role in inhibiting the development of malignancies [5, 9, 25].

In addition to these general effects, EGCG has also been shown to affect several cellular and molecular targets in the signal transduction pathways associated with cell death and cell survival. The precise descriptions of these cellular and molecular targets are mentioned later in “Section 4” of this study. The inhibition and/or activation of these targets by EGCG finally induce apoptosis and cell cycle arrest in both damaged and cancer cells, thereby demonstrating a beneficial clinical effect [9–11]. For instance, in current clinical trials, EGCG and Polyphenon E (Poly E), a standardized and well-characterized decaffeinated extract of green tea, have been shown to demonstrate an anti-carcinogenic effect in the patients with HPV-infected cervical lesions [33]. Another particularly encouraging study was a clinical trial for prostate cancer chemoprevention with oral green tea catechins [34]. It is also important to mention that no significant side effects have so far been noted to be associated with the consumption of the green tea catechin preparations at the daily doses that were administered in these studies.

## 3. Membrane Associated RTKs, their Downstream Signaling Pathways, and Transcription Factors

**Figure 2.**
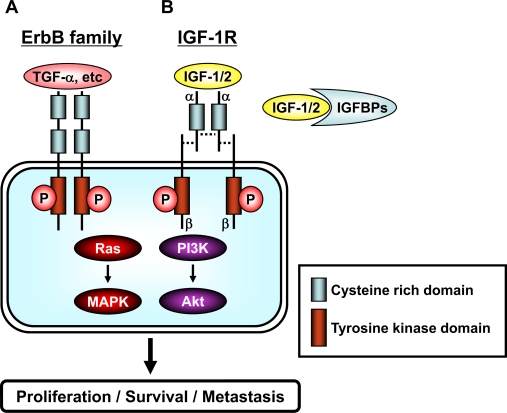
Schematic representation of the erbB family and the IGF/IGF-1R system. (*A*) The family of erbB receptors includes four members: EGFR (erbB1), HER2 (neu/erbB2), HER3 (erbB3), and HER4 (erbB4). All members have an extracellular ligand binding region (cysteine rich domain), a single membrane-spanning region, and a cytoplasmic tyrosine-kinase-containing domain. Ligand, such as TGF-α etc, binding to erbB receptors induces the formation of receptor homo- and heterodimers and the activation of the intrinsic kinase domain, thus resulting in phosphorylation on specific tyrosine residues within the cytoplasmic tail. These phosphorylated residues serve as docking sites for a range of proteins, the recruitment of which leads to the activation of intracellular signaling pathways, including the Ras/MAPK and PI3K/Akt pathways. (*B*) The IGF/IGF-1R system is composed of ligands (IGF-1 and IGF-2), receptor (IGF-1R), and ligand binding proteins (IGFBPs). IGF-1 and IGF-2 are found in the circulation complexed to IGFBPs, which serve to regulate the bioavailability of these ligands in the tissues. The IGF-1R contains two α (cysteine rich domain) and two β (tyrosine kinase domain) subunits which are joined by disulfide bridges to form a heterotetrameric receptor complex. The IGF/IGF-1R interaction results in phosphorylation of tyrosine residues in the tyrosine kinase domain. After autophosphorylation, the receptor kinase phosphorylates intracellular proteins, which enable activation of the PI3K/Akt and Ras/MAPK signaling pathways. A detailed description of the downstream signaling pathway is provided in “Figure 3”.

The activation of membrane-associated RTKs located on the cell surface by specific ligands (growth factors and cytokines) has been shown to play an important role in the control of many fundamental cellular processes (Figure 2) [21, 22, 35]. The EGFR (erbB1), HER2 (neu/erbB2), HER3 (erbB3), and HER4 (erbB4) proteins belong to subclass I (erbB) of the RTK superfamily (Figure 2*A*) [19, 21, 22]. IGF-1R is also a membrane associated RTK (Figure 2*B*) [20, 21]. All family members of RTK have an extracellular ligand-binding domain, a membrane-spanning region and a cytoplasmic protein tyrosine kinase domain. The EGFR is activated by TGFα, EGF, and other ligands, but no specific ligand for HER2 has yet been identified [22]. A family of ligands binds the extracellular domain of erbB receptors, thus leading to the formation of both homo- and heterodimers. Dimerization consequently stimulates the intrinsic tyrosine kinase activity of the receptors and triggers the autophosphorylation of specific tyrosine residues, thereby phosphorylating and activation the downstream pathways, including Ras/MAPK and PI3K/Akt pathways. HER2 is the preferred heterodimerization partner for the other members of the erbB family of RTKs (Figure 2*A*) [22]. The IGF-1R has two major ligands, IGF-1 and IGF-2, and binding of ligands to its receptor results in intramolecular receptor autophosphorylation and phosphorylation of cellular substrates that consequently lead to activation of distinct signaling pathways, including PI3K/Akt pathway and Ras/MAPK pathway, thus inducing gene activation, DNA synthesis and cell proliferation (Figure 2*B*) [20, 21]. Ultimately, the downstream effects on gene expression determine the biological response to receptor activation.

The transcription factors AP-1 and NF-κB are critical downstream effectors of the Ras/MAPK and the PI3K/Akt signaling pathways, respectively. AP-1 is a protein dimer composed of members of the Jun and Fos families. The binding of the AP-1 complex to the TRE sequence present in the promoter region of several genes is induced by growth factors, cytokines, and oncoproteins, and a high AP-1 activity is involved in the tumor promotion and progression of various types of cancers [41]. NF-κB is a sequence specific transcription factor that is known to be involved in inflammation, innate immunity, growth, and cell death (apoptosis). Because of these fundamental effects of the NF-κB pathway on normal cell physiology, the abnormality of this pathway also plays an important role in the development of various types of cancer [42]. The findings of reports therefore suggest that, in addition to RTKs and their downstream signaling pathways, the transcription factors AP-1 and NF-κB are thus promising targets for the treatment and prevention of cancer [41, 42]. It is of great interest that, in recent studies, several phytochemicals that have antitumor activity suppressed the activation of RTKs and their downstream molecules in cancer cells [2,5]. This subject will be discussed in greater detail in the next section, especially focusing on the effects of EGCG.

## 4. Effects of EGCG on the erbB Family of RTKs

As mentioned in “Section 2”, EGCG has various anticancer effects, including the inhibition of oxidative stress, the inhibition of carcinogen-induced mutagenesis, the induction of apoptosis, and the inhibition of angiogenesis [9]. We and other investigators have so far focused our attention on the effects of EGCG on cell surface RTKs, especially for erbB family proteins, as a mechanism for these anti-cell proliferative effects. First, Liang *et al*. [43] demonstrated that EGCG directly blocks EGF binding to the EGFR and thus subsequently inhibits the tyrosine kinase activity of the receptor in human A431 epidermoid carcinoma cells. We recently extended this finding and found that EGCG inhibits the activation of EGFR, HER2, and HER3, and their multiple downstream signaling pathways in human HNSCC, breast cancer, and colon cancer cell lines [10, 11, 13, 15]. As a result, EGCG inhibits both the activation of ERK and the basal and TGFα-stimulated c-*fos* and cyclin D1 promoter activity, while also causing a decrease in the cellular levels of the cyclin D1 and Bcl-x_L_ proteins. This effect on cyclin D1 may explain why the treated cells were arrested in G_1_ and the effect on Bcl-x_L_ may thus contribute to the apoptotic effect of EGCG [13, 15]. In the HT29 human colon cancer cells, EGCG and Poly E inhibit the activation of EGFR and HER2, the phosphorylation of Akt and ERK proteins, and also the transcriptional activity of the AP-1 and NF-κB promoters [10]. Similar effects are also observed in the SW837 human colon cancer cell line that expresses a high level and constitutive activation of HER3 while also demonstrating a high level of COX-2 protein. Therefore, EGCG inhibits the activation of EGFR, HER2, while also inhibiting the HER3 signaling pathways, the expression of COX-2 at the levels of transcription, and the production of PGE_2_, a major metabolic product of COX-2, by these cells [11].

Other investigators found that EGCG inhibits the tyrosine phosphorylation of HER2 in a mouse mammary tumor cell line and this was associated with the inhibition of the PI3K/Akt kinase and NF-κB signaling pathways [44]. Although no direct ligand for HER2 has yet been discovered, there is increasing evidence that HER2 is the preferred heterodimerization partner for the other members of the erbB family of RTKs [22, 45]. In addition, amongst the members of the erbB family, HER3 is the most efficient activator of PI3K since it contains six docking sites for the p85 protein, an adaptor subunit of PI3K [22]. These findings suggest that, in addition to EGFR, HER2 and HER3 may both play a critical role in the potentiation of erbB receptor signaling. Therefore, agents like EGCG which target EGFR, HER2, and HER3 may thus provide an effective strategy for inhibiting the growth of cancer cells that display an overexpression and activation of these receptors.

## 5. Effects of EGCG on the IGF/IGF-1R and VEGF/VEGFR Systems

Moreover, there is increasing evidence that EGCG inhibits the tyrosine kinase activities of the other additional RTKs. For instance, EGCG inhibits the activation of PDGFR and FGFR in human A431 epidermoid carcinoma cells [43]. We recently reported that EGCG inhibits the activation of IGF-1R in SW837 human colon cancer and HepG2 human liver cancer cells that display a constitutive activation of this receptor [12, 16]. The IGF/IGF-1R system, which includes the ligands IGF-1/2, their receptor IGF-1R, and the ligand controlling proteins IGFBPs, plays an important role in the development and growth of various types of cancer, especially prostate, colorectal, and liver cancer, and thereby may be a useful target for the prevention and treatment of cancers [20]. In these studies, EGCG causes a decrease in the expression levels of IGF-1/2, but an increase in the expression levels of IGFBP-3, which negatively controls the expression of IGF-1/2, in colon and liver cancer cells [12, 16]. EGCG has also been reported to induce apoptosis and inhibit the activation of ERK and Akt proteins both in the absence or presence of ligand stimulation [16]. Other investigators have demonstrated that the oral infusion of a green tea polyphenol mixture inhibited the development and progression of prostate cancer in a mouse transgenic model (TRAMP) of this disease [46]. This was associated with a reduction in the levels of IGF-1, a decreased activation of Akt and ERK, and decreased levels of VEGF and MMPs-2 and -9 in the dorso-ventral prostate of these mice [46]. These findings provide another example of the ability of EGCG to inhibit the RTK-related pathways of signal transduction.

In addition, in human HNSCC and breast cancer cell lines EGCG inhibits the constitutive activation of the transcription factor Stat3, which also lies downstream of EGFR [13–15]. Furthermore, EGCG inhibits the production of VEGF in human HNSCC and breast cancer cells, apparently by inhibiting both the activation of Stat3 and NF-κB in these cells [14]. VEGF is a mitogen for endothelial cells which is often associated with tumor-induced angiogenesis and the biological effects of VEGF are mediated by two RTKs, namely VEGFR-1 and -2, [47, 48]. Neuhaus *et al*. [49] demonstrated that EGCG inhibits VEGF-induced DNA synthesis, cell proliferation, the autophosphorylation of VEGFR-1 and -2, as well as phosphorylation of ERK protein in human umbilical arterial endothelial cells. As a result, these effects may contribute to the anti-angiogenic effects of EGCG.

## 6. Direct Effects of EGCG on Signaling Pathways and Transcription Factors

In addition to the effects of EGCG on cell surface RTKs, there is evidence that EGCG can directly target intracellular signaling pathways and transcription factors. For instance, a detailed study in immortalized human cervical cells found that EGCG inhibited the activation of EGFR, ERK, and Akt proteins, but this is not solely due to a reduction in the activity of initial kinase EGFR. These investigators therefore demonstrate that EGCG can directly inhibit the subcellular kinase activity of ERK and Akt [50]. In H-*ras* transformed mouse epidermal cells, EGCG directly causes a decrease in the activation of ERK and MEK1, a decrease in the association of Raf-1 with MEK1, and the inhibition of the AP-1 activity [51]. In addition, in subcellular assays EGCG was shown to directly inhibit the phosphorylation of Elk-1 by phospho-ERK, apparently by interfering with the binding of the Elk-1 protein substrate to the phospho-ERK kinase substrate [51]. EGCG thus inhibits the malignant transformation induced by EGF or TPA in the mouse epidermal JB6 cell line and this effect may be mediated, at least in part, by the direct inhibitive effect of EGCG on the activation of AP-1-dependent and NF-κB sequence-specific DNA-binding activity in these cells [52, 53]. EGCG also caused a significant inhibition of UVB-mediated activation of IKKα, degradation and phosphorylation of IκBα, and the nuclear translocation of the NF-κB transcription factor in human epidermal keratinocytes [54]. These findings suggest that it is incorrect to assume that the inactivation of the initial kinase in a cascade will result in the inactivation of all downstream responses and therefore the anti-tumor effects of EGCG may be due to the binding to multiple cellular targets.

## 7. Effects of EGCG on Expression Levels of Cyclin D1 and COX-2

The activation of AP-1 and NF-κB can act independently, and/or coordinately, to regulate the expression of specific target genes, including the *cyclin D1* and *COX-2* genes, since the promoter region of these genes contain binding sites for both AP-1 and NF-κB [55–57]. It is interesting to note that the cyclin D1 expression is required for breast carcinogenesis following mammary-specific expression of the HER2 or H-*ras* oncogenes [58]. HER2 and/or H-*ras* also trigger signaling cascades that lead to NF-κB activation in breast cancer [59, 60]. The NF-κB signaling pathway, which is activated by the Akt pathway, plays an important role in regulating COX-2 expression via the NF-κB binding site in the COX-2 promoter [61]. In colon cancer cells, the activation of HER3 by its ligand heregulin strongly induces COX-2 expression and PGE_2_, thus leading to an increase in cell proliferation and motility via activation of the PI3K/Akt pathway [62, 63]. Both we and other investigators found that several phytochemicals, including EGCG, that have antitumor activity can suppress the activation of AP-1 and NF-κB, while also inhibiting the expression of target molecules cyclin D1 and COX-2 in cancer cells [2, 10, 11, 13, 15]. Therefore, the inhibition of the cyclin D1 and COX-2 expression may also contribute to the growth inhibitory effects of EGCG. Similar effects of EGCG and Poly E have also been observed in *in vivo* studies. Therefore, treatment with these agents significantly attenuates the inflammation-related mouse colon carcinogenesis by reducing the expression of COX-2 protein and mRNA [32]. These findings are significant because chronic inflammation raises the risk for the development of some types of human malignancies, including colorectal cancer.

## 8. Lipid Rafts: a New Target of EGCG

The above studies provide evidence that EGCG can inhibit the pathways of signal transduction and gene expression that play critical roles in both carcinogenesis and tumor growth. This review focused mainly on the inhibitory effects of EGCG on the erbB family and IGF-1R of RTKs and their downstream signaling pathways, and the inhibitory effects of this chemical on the transcription factors AP-1 and NF-κB, which normally stimulate cell proliferation and enhance cell survival, because we have emphasized these aspects with respect to the antiproliferative mechanisms of EGCG [5, 10–16]. As previously described in this review, the members of the RTKs are activated by their specific ligands and then EGCG can directly inhibit the binding of these ligands, including EGF, PDGF, and FGF, to their respective receptors [22, 43, 64]. This could account for the inhibitory effects of EGCG on the autophosphorylation of the EGFR, HER2, and HER3 receptors in various types of cancer cells [10, 11, 13, 15, 43, 44]. Indeed, time course studies in human colon cancer cells have demonstrated this effect to occur within 6 hours, while the inhibition of the phosphorylation of the ERK and Akt proteins occurs after about 6 to 12 hours [10, 11]. These data suggest that EGCG initially inhibits the activation of RTKs on the cell surface, thus leading to the subsequent inhibition of the activation of downstream effectors in the cytoplasm, including the ERK and Akt proteins.

On the other hand, as mentioned in “Section 6”, there is evidence that EGCG can directly target the intracellular signaling molecules [50, 51]. Taken together, these findings suggest that the antitumor effects of EGCG may be due to binding, probably with a relatively low affinity, to multiple cellular targets. However, the precise identification of the direct and critical cellular target(s) of EGCG still remains to be determined. In a recent study the 67-kDa laminin receptor (67-LR), which is closely associated with lipid rafts, was interestingly shown to confer EGCG responsiveness to cancer cells at physiologically relevant concentrations (0.1 to 1.0 μM). Therefore, the authors demonstrated that the lipid raft-associated 67-LR plays an important role in mediating the action of EGCG [65–67]. The IGF-1R and the members of erbB family are also localized in lipid rafts [68–70]. The localization of the erbB family to lipid rafts appears to modulate both their ligand binding and tyrosine kinase activities, while the lipid environment in the cell membrane profoundly influences their association properties and biological functions in epithelial cancer cells [68, 71, 72]. Adachi *et al*. [17] recently reported that EGCG causes a reduction in ordered lipid domains in the plasma membrane of colon cancer cells and this is associated with inhibition of activation of the EGFR and related downstream signaling pathways in these cells. These findings suggest that EGCG may exert its biological effects primarily through, at least in part, the cell membrane lipid rafts.

## 9. Future Perspectives: Possibility for Clinical Application of EGCG

A critical issue when agents are applied as chemopreventive drugs in clinical use is whether a specific agent can exert its anti-cancer effects without causing any undesirable side effects, *i.e.*, whether the agent can selectively or preferentially inhibit pre-neoplastic or cancer cells without significantly affecting the normal cells in the host. With respect to this aspect, it is encouraging that, in the numerous rodent studies with EGCG, anti-cancer effects were observed with little or no toxicity to the host animals [9]. A possible explanation for this is that the roles of specific RTKs and the complex circuitry of the signal transduction pathways in cancer cells differ sufficiently to provide differential responses to these compounds [73]. For instance, Ahmad *et al*. [74] found that EGCG causes an inhibition of cell growth and the induction of apoptosis in human epidermoid carcinoma cells, while causing no such effects in normal human epidermal keratinocytes. The authors suggested that this was due to the fact that EGCG inhibits the activation of NF-κB in carcinoma but not in the normal cells [74]. EGCG preferentially inhibits the growth of human colon cancer cells in comparison to FHC normal human fetal colon cells, and this phenomenon is associated with the constitutive activation of the EGFR and HER2 proteins in colon cancer cells but not in normal colon epithelial cells [10]. EGCG also preferentially inhibits the growth of HepG2 human hepatoma cells, which highly express the IGF-1R proteins, in comparison to Hc normal human hepatocytes, which less express of this protein [16]. In addition, a relatively low concentration of EGCG, 1.0 μg/ml, which is almost the same level as the peak plasma concentration of EGCG in clinical trials [75], can inhibit the growth and the activation of EGFR HER2, and HER3 receptors, thereby inhibiting the expression of COX-2 and inducing apoptosis in colon cancer cells when the cells were exposed to this agent for long periods of time [10,11].

Similar results are also conformed *in vivo* studies. Thus, we found that not only a high (0.1%), but also a low (0.01%) concentration of EGCG similarly decrease the development of colonic adenocarcinoma in inflammation-related mouse colon carcinogenesis [32]. Furthermore, drinking a low as well as high doses of EGCG can inhibit the development of colonic premalignant lesions by improving the metabolic abnormalities to the same extent observed in obesity-related mouse colon carcinogenesis (unpublished data). These reports indicate that a low dose of EGCG (0.01%) is sufficient to prevent the development of colon tumor, thus suggesting the possibility that the EGCG dose thus can be further decreased, although the feeding protocol of EGCG at a high dose (0.1%) mimics an approximate consumption of 6 cups of green tea per day by an average adult human and has been used in mice in many prior chemopreventive studies [9]. These findings, therefore, seem to be very encouraging and are even more significant when considering the clinical use of this agent, because a reduction in the dosage of the agent is important to reduce the incidence of adverse effects, especially in daily life, and is thus more acceptable for administration to patients.

Indeed, a recent double-blind, placebo-controlled study with green tea in Italian patients demonstrated a successful prevention without causing adverse effects. Thus, in the trial, the progression of prostate cancer in men with high-grade prostate intraepithelial neoplasia, the main premalignant lesion of prostate cancer, was significantly prevented by oral administration of green tea catechins (GTCs) [76]. Based on our findings that EGCG possess anti-cancer effects in colorectum [10, 11, 12, 32], we are now conducting a randomized trial of GTCs for the prevention of metachronous colorectal adenomas in subjects of a high risk group, *i.e.*, those with post-polypectomy state of their preceding colorectal adenomas, while focusing on the effects of GTCs on the activation of specific RTKs in the colonic mucosa.

## 10. Conclusions

This review presents evidence that the inhibitory effects of EGCG on carcinogenesis are mediated, at least, through the regulation of some members of RTKs and cell signaling pathways, whereas other mechanisms also play a role in inducing an anti-cancer effect [9]. Finally, we would like to emphasize that, in current studies, it is very significant to design and develop chemopreventive agents that act on specific molecular and cellular targets. We also therefore believe that EGCG may be a potent phytochemical because of its specific effect on targeting both RTKs and multiple pathways.

## Figures and Tables

**Figure 3.**
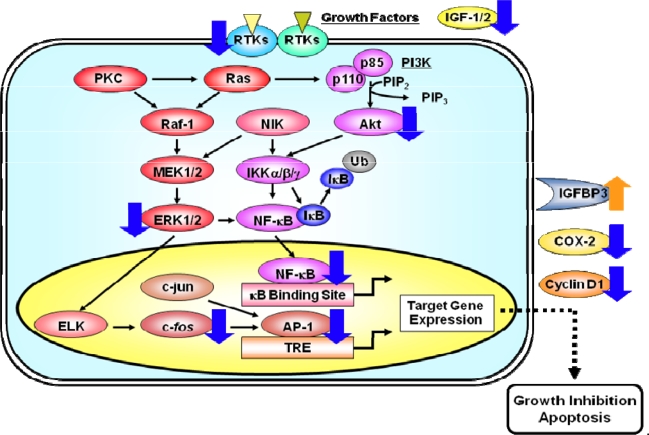
A simplified scheme indicating how the activation of RTKs induces pathways of signal transduction which lead to the activation of the transcription factors AP-1 and NF-κB. RTKs, like EGFR and IGF-1R, are activated by specific ligands, thus leading to the activation of their intrinsic tyrosine kinase and autophosphorylation of tyrosine residues. These activated RTKs then phosphorylate several downstream molecules, thus activating several signaling pathways. The activation of the small G protein Ras and effector proteins, such as Raf-1 and PI3K, stimulates several intracellular processes. As a result, the activated Raf-1 stimulates the MEK1/2 and its cascade which then phosphorylate the MAPK protein ERK1/2. Once activated, MAPKs can activate a variety of transcription factors, including ELK and c-Jun. The binding of AP-1, a dimeric complex that comprises members of the Jun and Fos families of transcription factors, to the TRE DNA sequence in various gene promoters, activates the expression of target genes. PI3Ks are heterodimeric lipid kinases that are composed of a regulatory (p85) and a catalytic (p110) subunit. The activation of PI3K causes the synthesis of the lipid PIP_3_, which activates the downstream pathways that involve Akt. Akt and NIK both play a role in the phosphorylation and activation of the kinase IKK. Activated IKK phosphorylates IκB, which triggers ubiquitinylation (Ub) and the subsequent degradation of IκB. The loss of IκB releases Rel from an inactive complex, which then translocates from the cytoplasm to the nucleus where it can activate the transcription of target genes. Molecules that appear to be cellular targets for the action of EGCG are indicated by blue (downregulation) or orange (upregulation) arrows. These multiple effects of EGCG finally lead to the inhibition of cell proliferation and the induction of apoptosis in cancer cells
